# Exercise-Stimulated ROS Sensitive Signaling Pathways in Skeletal Muscle

**DOI:** 10.3390/antiox10040537

**Published:** 2021-03-30

**Authors:** Jessica Bouviere, Rodrigo S. Fortunato, Corinne Dupuy, Joao Pedro Werneck-de-Castro, Denise P. Carvalho, Ruy A. Louzada

**Affiliations:** 1 Institut of Biophysics Carlos Chagas Filho, Federal University of Rio de Janeiro, Rio de Janeiro 21941-902, Brazil; jessicabouviere1@gmail.com (J.B.); rodrigof@biof.ufrj.br (R.S.F.); dencarv@biof.ufrj.br (D.P.C.); 2 Université Paris-Saclay, UMR 9019CNRS, Gustave Roussy, 94800 Villejuif, France; corinne.dupuy@gustaveroussy.fr; 3 Division of Endocrinology, Diabetes and Metabolism, Miller School of Medicine, University of Miami, Miami, FL 33136, USA; j.werneckdecastro@med.miami.edu

**Keywords:** redox signaling, ROS, exercise, NADPH oxidase, skeletal muscle, oxidative stress, reductive stress

## Abstract

Physical exercise represents a major challenge to whole-body homeostasis, provoking acute and adaptative responses at the cellular and systemic levels. Different sources of reactive oxygen species (ROS) have been described in skeletal muscle (e.g., NADPH oxidases, xanthine oxidase, and mitochondria) and are closely related to the physiological changes induced by physical exercise through the modulation of several signaling pathways. Many signaling pathways that are regulated by exercise-induced ROS generation, such as adenosine monophosphate-activated protein kinase (AMPK), mitogen activated protein kinase (MAPK), nuclear respiratory factor2 (NRF2), and PGC-1α are involved in skeletal muscle responses to physical exercise, such as increased glucose uptake, mitochondriogenesis, and hypertrophy, among others. Most of these adaptations are blunted by antioxidants, revealing the crucial role played by ROS during and after physical exercise. When ROS generation is either insufficient or exacerbated, ROS-mediated signaling is disrupted, as well as physical exercise adaptations. Thus, an understanding the limit between “ROS that can promote beneficial effects” and “ROS that can promote harmful effects” is a challenging question in exercise biology. The identification of new mediators that cause reductive stress and thereby disrupt exercise-stimulated ROS signaling is a trending on this topic and are covered in this current review.

## 1. Introduction

Physical exercise represents a major challenge to whole-body homeostasis, provoking widespread perturbations in cells, tissues, and organs. Several responses, which are generally reversible, take place at local and systemic levels during and after acute physical exercise, which require a permanent stimulus that is provided by regular exercise training [1].

The beneficial effects of exercise are related to the activation of multiple integrated and occasionally redundant cellular signaling pathways, which occur in the muscle and in other tissues simultaneously [1,2,3]. Recent discoveries about the mechanisms by which a contracting muscle ‘‘communicates’’ with other organs are emerging as an exciting new field of research that reaffirms the essential importance of studies in exercise biology [3]. For a long time, reactive oxygen species (ROS) generated during exercise were believed to have deleterious effects on health. However, a new concept has been evolving over the last 20 years that identifies ROS as critical mediators of signal transduction pathways, but there are still several questions to be answered (for more historical details see [4,5]).

ROS, such as superoxide (O_2_^•−^), hydroxyl (OH^•^), and the non-radical species, H_2_O_2_, are small radical or non-radical molecules that can react with a wide spectrum of molecules, changing their structures in a reversible or irreversible way and modifying their function [6]. ROS are implicated in the pathophysiology of diseases and in important physiological mechanisms that are related to redox signaling, hormonal biosynthesis, and pathogen elimination [7]. ROS generation is antagonized by cellular antioxidant activity, which functions to prevent, delay, or remove oxidative damage caused by biological molecules. The antioxidant mechanisms may be enzymatic (e.g., superoxide dismutase, glutathione peroxidase, catalase, and peroxiredoxins) or non-enzymatic (e.g., thioredoxins, vitamins C and E). Therefore, the availability of ROS and the extent of their effects depend on a fine balance between their generation and elimination. Thus, an imbalance between oxidants and antioxidants in favor of the oxidants, leading to a disruption of redox signaling and control and/or molecular damage is called oxidative stress [8]. Furthermore, the ability of exercise training to induce beneficial health effects [9] is prevented when ROS-mediated signaling is disrupted by antioxidants [10], which was recently reinforced in a meta-analysis of randomized controlled trials [11].

Several different strategies using cutting-edge technology have been used to decipher the role of ROS in exercise physiology, especially in within contracting muscle [12]. In addition, a rich understanding of responses and adaptation induced by exercise that are mediated by redox reaction have been outstandingly discussed [13]. Particularly, in our previous review [3], we discussed how contracting muscle communicates to the whole body to promote the widespread health benefits of exercise. We focused on metabolites, cytokines, and lipid peroxidation as the mechanisms by which exercise creates the ROS-rich environment in contracting and non-contracting cells. Herein, we discussed the main ROS sources and highlighted several well-defined mechanisms and some other potential candidates to promote burst of ROS following contraction. In addition, we revisited the concept of regulating kinase and phosphatase tonus by ROS through specific modifications of key targets (cysteines/proteins) during exercise. Lately, the identification of a hepatokine that caused reductive stress and disrupted exercise-stimulated ROS signaling is discussed, suggesting that reductive stress is likely to be as crucial as any muscle-specific deletion of ROS source. Finally, we underlined the ambivalent role of NOX2 in muscle either in the health benefits of exercise or in the pathophysiological conditions.

## 2. Main Sources of ROS in Skeletal Muscle during Contraction

**Figure 1:**
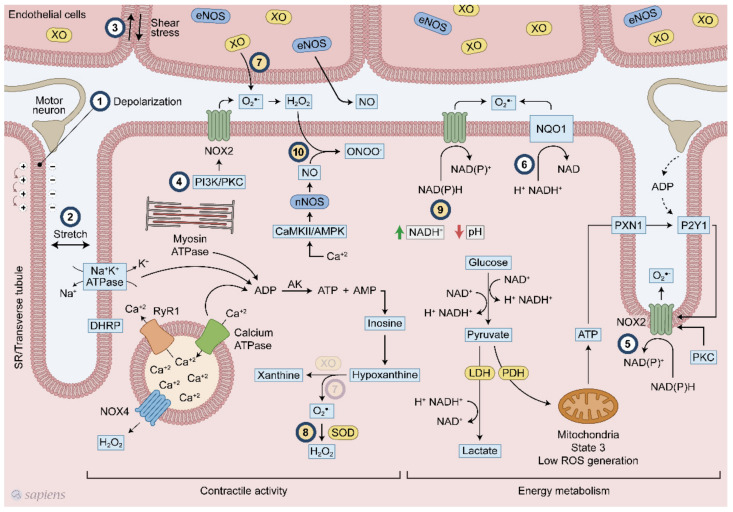
Overview of the mechanisms of reactive oxygen species (ROS) generation following exercise. Multiple mechanisms related to ROS generation in skeletal muscle during contraction. #1 membrane depolarization, #2 stretch #3 shear stress, #4 PI3K/PKC, #5 ATP/P2Y1, #6 NQO1 #7 XO, #8 SOD, #9 NADH/pH and #10 CaMK/AMPK. Numbers from 1 to 6 (#1–6) indicate signaling pathways already established. Numbers from 7 to 10 (#7–10) show hypothetical pathways. #7-The presence of XO in skeletal muscle is still a debated topic.

Skeletal muscle has several sources of ROS, but mitochondria [14], NADPH oxidase enzymes (NOX/DUOX) [15], and xanthine oxidase (XO) [16] seem to be the three most relevant during exercise. While O_2_^•−^ are generated by mitochondria and XO as a secondary sub-product of oxidative phosphorylation and purine metabolism, respectively, NAPDH oxidase enzymes are exclusively dedicated to generating superoxide and H_2_O_2_ [17,18] (Figure 1).

ROS are generated by skeletal muscle at rest, and during contractile activity, they increase significantly in the intracellular medium and in the muscle interstitial space [19,20]. ROS production during skeletal muscle contraction and physical exercise has been the subject of considerable discussion over the last 40 years. The labile nature of ROS and methodological difficulties in detecting these molecules are still present, but remarkable advances have been made in the recent years, mainly regarding the detection of subcellular ROS generation through biosensor redox-active compounds and an improvement of available image techniques [21,22,23]. The use of genetically encoded redox probes has been crucial to the recent advances of the ROS field. Recently, Henriquez-Olguin et al. were able to isolate the cytoplasmic from the mitochondrial ROS production in vivo using NOX2-specific ROS production biosensor of human p47phox fused to the N terminus of redox-sensitive green fluorescent protein (p47roGFP) and Mito-roGFP2-Orp1 probe respectively [24]. In addition, formalin-fixed paraffin-embedded muscle can be analyzed by the optical redox imaging (ORI) technique that was suitable to measure cellular autofluorescence of nicotinamide adenine dinucleotide (NADH) and oxidized flavoproteins, where its ratio indicates the redox state in soleus and gastrocnemius muscles [25]. Those strategies allow to follow ROS generation in different cell compartments simultaneously and in several experimental conditions. In this way, the temporal and spatial fashion of ROS production and redox state can be measured.

Mitochondrial complex I and II are the main sites of O_2_^•−^ production in the electron transport chain [26]. Initially, it was believed that the mitochondria were the main source of O_2_^•−^ in skeletal muscle, with reports showing that between 2% and 5% of the oxygen consumed by mitochondria was converted to O_2_^•−^. However, more recent data do not support this evidence, showing that the rate of O_2_^•−^ production from molecular oxygen in the mitochondria is about 0.15% [4]. Skeletal muscle mitochondria produce more O_2_^•−^ in the basal condition than during physical exercise. In the basal condition, mitochondria operate at respiration state 4. However, during exercise, the mitochondria operate at state 3 (which is also known as the maximal ADP stimulated respiration) where O_2_^•−^ generation is lower [14], even when either succinate or pyruvate/malate are used as a substrate [27,28]. Additionally, when mitochondria are exposed to an exercise condition, H_2_O_2_ production is significantly reduced, indicating that they are not the main source of ROS following exercise [26]. Recently, this reduction was also observed in mice after 20 min of treadmill exercise measured by oxidation of Mito-roGFP2-Orp1 probe [24]. Therefore, it has been proposed that NOX enzymes play important physiological roles in skeletal muscle and they are extremely relevant in muscle excitation-contraction coupling [15,29] and in the modulation of signaling pathways that are involved in exercise training adaptation [30].

The NOX family is composed of seven members, NOX1 to NOX5 and DUOX1 and 2, which are differentially expressed among tissues. NOX1, 2, and 4 and DUOX1/2 have been reported to be present in skeletal muscle [15,31,32]. The unique function of NOX enzymes is ROS production. NOX1 and 2 produce O_2_^•−^, while DUOX1/2 and NOX4 generate H_2_O_2_. DUOXs have EF hand motifs in their cytoplasmic domains that are related to the regulation of their activities by calcium. Moreover, both targeting to the plasma membrane and functioning of DUOX enzymes require maturation factors known as DUOX activator 1 and 2 (DUOXA1/A2) [17,33,34]. NOX2 is constitutively associated with the protein p22phox in the biological membrane. Activation of the NOX2-p22phox complex requires the translocation of cytosolic factors such as p47phox, p67phox, and p40phox to the membrane. Phosphorylation of p47phox induces a change in its conformation that enables it to bind p22phox, thus promoting the interaction of the other cytosolic factors with NOX2 [18]. Additionally, p22phox is a functional binding partner of NOX4, but its activity seems to be mainly dependent on its expression level, as well as the partial oxygen pressure [17,29,35]. Moreover, ATP can directly bind and negatively regulate Nox4 activity in the inner mitochondrial membrane, suggesting that the subcellular redistribution of ATP levels from the mitochondria might act as an allosteric switch to activate NOX4 [36].

The relative contribution of mitochondrial and nonmitochondrial ROS was investigated in isolated fibers from mouse flexor digitorum brevis in two independent studies [15,37]. In both studies, NADPH oxidase rather than mitochondria appears to be the major contributor of cytosolic ROS in skeletal muscle. NADPH oxidase inhibitors decreased cytosolic ROS at rest and following contraction. NOX2, NOX4, and p22phox were found in sarcolemma and transverse tubules of isolated fibers, whereas the other partners such as p40Phox and p67phox protein that were found in cytoplasm were translocated to sarcolemma during muscular contraction [18]. NOX4 was also detected in mitochondria [15]. This was probably the first study that demonstrated the expression of NADPH oxidases and their partners in an isolated muscle fiber preparation, avoiding misinterpretation because of the presence of other cell types. Additionally, apocynin, a non-specific NOX inhibitor, prevented p47phox and p67phox translocation to the cell membrane and contraction induces superoxide production [15], corroborating previous observation that DPI, a non-specific NOX inhibitor, prevented the increased ROS production induced by contractile activity [37]. These data suggest that NOX2 is likely to be the predominant oxidase system that contributes to cytosolic superoxide generation in muscle fibers during contraction.

## 3. Intracellular Mechanisms Potentially Related to ROS Production Following Muscular Contraction

ROS production can be stimulated by several ways in skeletal muscle following exercise. Plasma membrane depolarization elicited by motoneuron electrical stimulation is the first step of muscle contraction (Figure 1, #1), and seems to stimulate NOX2 activity [15,38,39]. After sarcolemma depolarization, ATP is released with subsequent activation of P2Y1 receptor and PKC, which in turn activates NOX2 (Figure 1, #5) [40,41,42]. Additionally, skeletal muscle stretching stimulates the assembly of NOX2 with its regulatory subunits at the plasma membrane, increasing extracellular ROS production (Figure 1, #2) [42,43]. PI3K and PKC also activates NOX2 (Figure 1, #4) [18,40,44].

A higher energetic metabolism demand following physical exercise might be coupled with exercise-induced ROS production through cytoplasmatic glucose metabolism and NADH regeneration, as well as high ATP hydrolysis rates and purine metabolism. Lactate is formed in an intensity-dependent manner and it is released mainly by fast-twitch fibers, which have high amounts of glycogen and glycolysis as their main ATP source [45]. For glycolysis, during dehydrogenase enzymatic reaction, electrons are transferred from glycolysis metabolite to NAD^+^, forming NADH^+^ and H^+^. NAD^+^ regeneration is a limiting step for maintaining high ATP formation via glycolysis. For this purpose, muscle cells perform NAD^+^ regeneration through three main mechanism: (1) formation of lactate from pyruvate by the lactate dehydrogenase (LDH) enzyme; (2) mitochondrial shuttle involving glycerol phosphate and malate-aspartate [45]; and (3) NADH-cytochrome b5 oxidoreductase or NAD(P)H quinone oxidoreductase (NQO1) (Figure 1, #6) [46]. Although they are called NADPH oxidases because of their high affinity for NADPH, these enzymes are also able to use NADH as an electron donor in a less effective way. In phagocytes, NOX2 activity was detected with a NADH concentration of less than 0.8 mM while only 0.2 mM NADPH was required [47]. The affinity of NOX for NADH seems to be increased at low pH levels (Figure 1, #9) [47]. NADH levels after exercise increases considerably [48] in an intensity-dependent manner [49], reaching approximately 0.2 mM. In triads, which is the structure formed by a T tubule with a sarcoplasmic reticulum (SR), increased superoxide generation was observed either with the addition of 100 μM NADH or NADPH [39], Thus, one can speculate that during contraction, the increased intramuscular NADH levels originated from energetic metabolism could be used by NADPH oxidases to produce ROS. Recently, it was demonstrated that the conversion of lactate into pyruvate within intestinal cells generated NADH that was able to activate NADPH oxidases [50]. Moreover, the existence of an ATP-binding motif in NOX4 structure and its regulation by ATP [36,51] suggests that this enzyme can serve as a metabolic sensor potentially coupling exercise-induced ROS production to ATP metabolism.

XO has been recognized as contributing to superoxide generation in the extracellular space following muscle contraction [52,53]. The selective removal of O_2_^•−^ in rats [52] and human [54] has lighted the role of xanthine oxidase on exercise responses. XO is highly expressed in endothelial cells and one stimulatory mechanism suggested that muscle contraction alters the shear stresses applied to the skeletal muscle cell, which leads to O_2_^•−^ generation (Figure 1, #3). Moreover, XO can generate O_2_^•−^ through another mechanism that is linked to ATP hydrolysis. During skeletal muscle contraction, ATP hydrolysis is high mainly because of ATPase activity (e.g., myosin, SERCA, and the Na/K pump), which increases the ADP levels. Adenylate kinase (AK) is stimulated by ADP, which increases ATP and adenosine monophosphate (AMP) that is subsequently deaminated to inosine monophosphate (IMP). Inosine and hypoxanthine are produced from IMP, and then hypoxanthine is converted to xanthine by XO, generating superoxide (Figure 1, #7). Some established (#1–6) and hypothetical (#7–10) models of ROS production following muscular contraction are illustrated in Figure 1.

## 4. The Concept of ROS Modulating Phosphatases and Kinase Tone

**Figure 2:**
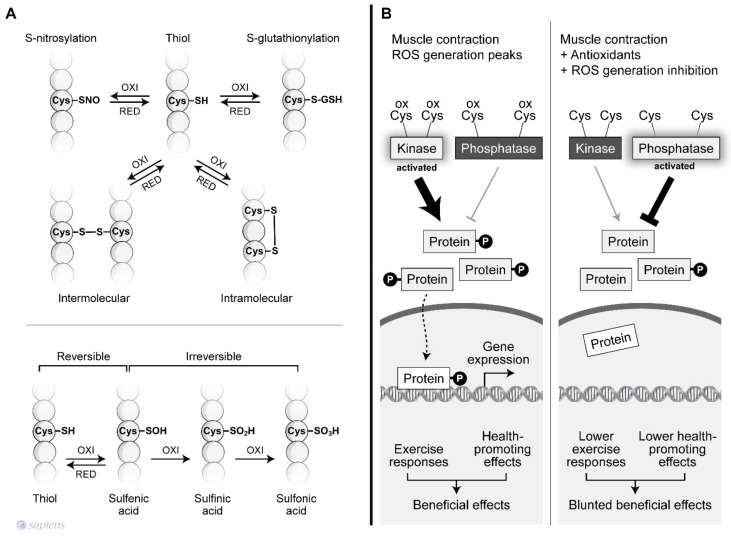
Redox-sensitive cysteine residues. (**A**) Thiol groups are vulnerable to oxidation and can lead to various post-translational modifications that are reversible or irreversible depending on the oxidation degree. Among then, the S-nitrosylation and S-gluthionylation alter the function of several proteins. The sulfhydryl (–SH) group may initially be reversibly oxidized to sulfenic acid (SOH), which is further oxidized to a sulfinic acid (SO_2_H) or irreversibly oxidized to sulfonic acid (SO_3_H). In addition, sulfhydryl groups on cysteines are the preferred targets for oxidation or formation of intra and intermolecular disulfide bonds. (**B**) ROS-mediated signaling orchestrates the final cellular response through the modulation of phosphatases and kinase tone. During muscle contraction, peaks of ROS decrease phosphatase activity and increase kinase activity, activating signaling pathways related to the beneficial effects of exercise. However, factors that disrupt ROS signaling during exercise can alter the kinase/phosphatase tonus leading to a blunted exercise response.

Redox-mediated signaling mainly occurs via the targeted modifications of specific residues in proteins [55]. Thus, the complex redox code behind the activation of specific exercise responses is not likely a “switch on/off” mechanism, but instead, redox modification is thought to occur, which affects the enzyme activity. The redox-based post-translational regulation of protein function occurs principally through modification of Cys thiol side chains within proteins. Sulfhydryl groups on cysteines are the preferred targets for oxidation or for the formation of disulfide bonds. The sulfhydryl (–SH) group may initially be reversibly oxidized to sulfenic acid (SOH), which is further oxidized to a sulfinic acid (SO_2_H) or irreversibly oxidized to sulfonic acid (SO_3_H). The hydroxyl radical can irreversibly oxidize protein sulfhydryl groups leading to protein damage (Figure 2A) [42,56].

During physical exercise, redox-sensitive protein modifications are related to an increase in the net phosphorylation levels, which activates a wide range of physiological responses. As documented by Wright et al., two groups of phosphatases, called tyrosine and serine/threonine phosphatases, are inhibited by H_2_O_2_ [57]. The previous conclusions that were extensively discussed by Barbieri et al. are revisited is this review to revalue this plausible hypothesis of “how redox-sensitive protein modification orchestrates many exercise responses” [58]. H_2_O_2_ was shown to inhibit all phosphatase activities, resulting in higher phosphorylation levels for a wide range of proteins in skeletal muscle [57]. Moreover, several protein kinases that are involved in exercise responses have redox-sensitive sites that, when oxidized, are related to an increase in kinase activity [59,60]. Figure 2B details how kinases and phosphatases are modulated by H_2_O_2_. Alternatively, SOD activity, which was initially placed into the enzymatic antioxidant group, has been described as mediating oxidation and phosphatase inactivation through the conversion of superoxide to H_2_O_2_, the most important ROS signaling molecule [61] (Figure 1, #8). Barbieri and Sertoli proposed an attractive hypothesis, suggesting that ROS might regulate “phosphatase and kinase tones”, which influence the kinetics and amplification of many signaling pathways (Figure 2).

## 5. Exercise-Generated ROS Activate Redox-Sensitive Transcription Factors

**Figure 3:**
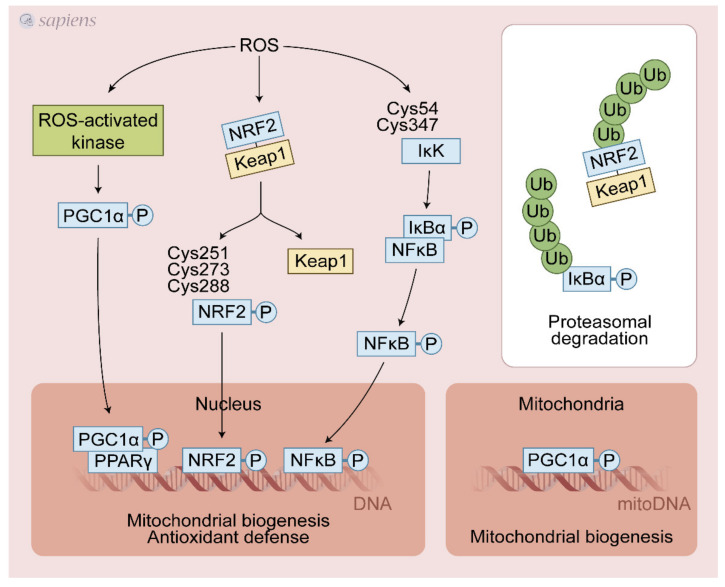
Exercise-generated ROS activate redox-sensitive transcription factors. ROS lead to various post-translational modifications that increase upstream pathways that promote PGC-1α activation (e.g., P38 MAPK, CaMK, AMPK). NRF2/Keap1 complex dissociation is stimulated by ROS, avoiding its proteasomal degradation thereby sustaining the NRF2 signaling. Additionally, ROS can directly activate NRF2 through cysteines modifications. NF-kB cysteines oxidation activates IkK that phosphorylates IkBα, leading to its dissociation and activation of NF-kB signaling. These redox-sensitive transcription factors regulate mitochondrial biogenesis and antioxidant defense in response to physical exercise.

ROS that are generated during contractile activity appear to both directly and indirectly mediate the activation of several redox-regulated transcription factors, including nuclear factor-kappa B (NF-κB), activator protein-1 (AP-1), heat shock factor-1 (HSF-1), nuclear factor erythroid 2 -related factor 2 (Nrf2), and peroxisome proliferator-activated receptor-γ coactivator-1α (PGC-1α) [10,62,63,64] (Figure 3).

NRF2 was recently identified as a key molecule in the transactivation of the antioxidant response element (ARE) and in the upregulation of several proteins involved in antioxidant defense and mitochondrial biogenesis in response to exercise. Under basal conditions, Keap1 targets Nrf2 for ubiquitination and proteolytic degradation, leading to rapid Nrf2 turnover [65]. ROS induced by physical exercise may promote the dissociation of the NRF2-Keap1 complex through the oxidation of cysteines 273 and 288 on Keap1 [66], avoiding the proteasomal degradation and then translocating NRF2 to the nucleus, activating the antioxidant-responsive element (ARE)-dependent gene and mitochondrial biogenesis [63]. ROS generated during contractile activity appear to activate NF-κB activity through oxidative modifications of Cys54 and 347 of IκK, which once activated, phosphorylates IκBα, leading to its dissociation from NF-κB. Then, NF-κB migrates to the nucleus and activates ARE [67] (Figure 3). The detailed source of ROS following exercise able to control NRF2 and NF-κB is still debated. Growing evidence points to a crucial role of NADPH over the mitochondrial source during exercise. NOX2 generated ROS (superoxide or H_2_O_2_) are likely involved in the activation of NRF2 and NF-κB. In addition, it has been proposed that PGC-1α can also activate NRF2 [68,69]. Interestingly, PGC-1α, NRF2, and NF-κB gene expressions were differently regulated in several experimental models of NOX2/NOX4 deletion in mice and pharmacological nonspecific inhibition of XO in rats. These studies were discussed in Section 7.1.

## 6. Exercise-Generated ROS Is Crucial to Muscle Glucose Uptake

Exercise is a highly effective strategy to decrease glucose blood levels mostly because of the capacity of contracting muscle to stimulate glucose uptake. Exercise can increase glucose transport to about 50-fold in humans [70]. Ca^2+^/calmodulin dependent kinase II (CaMKII) that is phosphorylated at Threonine 286 serves as a mediator of glucose uptake that is induced by muscle contraction. The calcium released with muscular contraction increases CaMKII activity that induces GLUT4 translocation and consequently glucose uptake. CaMKII is the most predominant CaMK isoform in skeletal muscle and its inhibition can reduce glucose uptake in vitro following contractile activity [71,72]. Oxidative modifications in the methionine pair 281/282, which are located in the regulatory domain of CaMKII [71,72], increase its activity, showing another potential mechanism that links ROS and glucose uptake during exercise.

**Figure 4:**
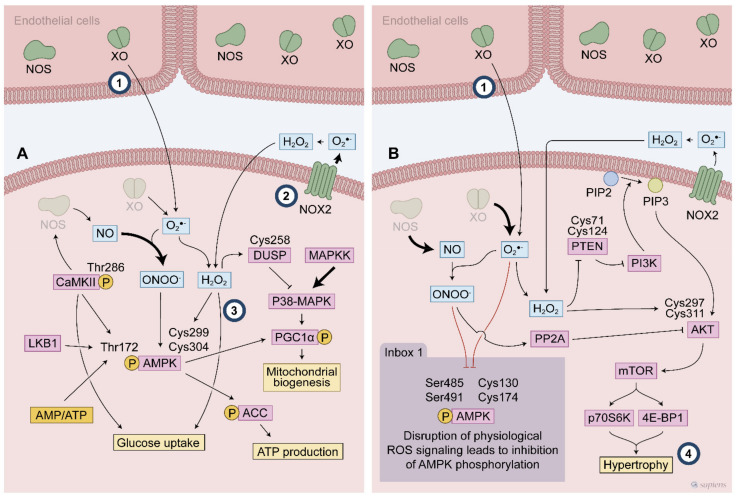
Exercise-generated ROS affect redox sensitive protein activated following endurance (**A**) and resistance exercise (**B**). During endurance exercise, the ROS mediated signaling activates multiple cascades such as P38 MAPK and AMPK to promote the exercise responses. ROS modify specific cysteines leading to AMPK phosphorylation at Thr172. AMPK activation is described to promote many exercise responses, such as glucose uptake and mitochondrial biogenesis. Dual-specificity phosphatase (DUSP) cysteine modifications is a possible target of ROS to control P38 MAPK signaling during exercise. High intensity exercise recruits many different ROS sources that contribute to this ROS mediated signaling. ROS participates of PI3K activation through both a direct mechanism involving AKT activation and an indirect mechanism by attenuation phosphatase activities (e.g., PP2A and PTEN), then favouring the hypertrophy signaling. Inbox: Thr 172 AMPK phosphorylation is blunted by disrupted ROS signaling where either an excessive or blunted ROS production alters the expected AMPK activation following exercise. (#1–4 indicates knowledge gaps. #1 indicates the debated topic about XO location. #2 the main source of ROS in contracting muscle. #3 the need to uncover the mechanisms by which exercise stimulates glucose uptake and #4 the involvement of PI3/Akt as a redox sensitive cascade to promote hypertrophy).

Regardless of exercise stimulation, upon insulin action, ROS generated by NOX is required for the intracellular Ca2+ increase that is mediated by the IP3 receptor, a process that is involved in the trafficking of vesicles containing GLUT4 to the plasma membrane [73,74]. Adding H_2_O_2_ directly to muscle cell increases glucose uptake through PI3K signaling pathway. Moreover, the pre-treatment with ROS scavengers (e.g., catalase and superoxide dismutase (SOD)) blunted the muscle glucose uptake that was induced by muscle contraction [70,75], showing that ROS produced during contractile activity is involved in glucose uptake. Despite the direct effect H_2_O_2_ on PI3K, the link between exercise generated ROS, PI3K activation, and consequently glucose uptake was not confirmed. Using isolated rat muscle, Wortmannin, an inhibitor of PI3K, did not prevent the contraction-stimulated glucose uptake [76,77] nor the GLUT4 translocation to the plasma membrane [76] showing that distinct signaling pathways are operating in skeletal muscle to stimulate exercise or insulin-stimulated glucose uptake. Treadmill exercise and experiments with isolated muscle from global deletion of Akt2 reinforced that exercise-stimulated glucose uptake is independent of the canonical signaling pathway triggered by insulin [78]. Intriguingly, the mechanisms involved in the exercise-stimulates glucose uptake are unknown and most notably, how ROS-sensitive mechanism operates to increase glucose uptake is still unexplored (#3 in Figure 4). In addition to increasing muscle glucose uptake, contractile activity increases hexokinase II, the rate-limiting enzyme of glycolysis [30]. Regardless the specific ROS source, antioxidants blunted the induction of HKII mRNA expression in electrically stimulated muscle cells [73,74] and training induced HKII mRNA in mice [79]. Physical exercise training increases HKII expression, which was abolished in NOX2-deficient animals. However, GLUT4 expression was not affected by the absence of NOX2 [30]. Interestingly, NOX2 was related to the regulation of glucose transport capacity during moderate-intensity exercise, once two NOX2 loss-of-function mouse models lacking either p47phox or Rac1 presented reduced exercise-stimulated glucose uptake and GLUT4 translocation [24]. There is a compartmentalization of ROS production [12,18] that is closely linked to the subsequent responses, which could explain why the same source of ROS is not linked to all exercise-induced adaptations.

Despite the lack of evidence about exercise-induced ROS on muscle glucose uptake via PI3K, eventually other mechanisms might be acting as coordinators of this effect. AMPK is a powerful metabolic sensor and was suggested a potential effector implicated in glucose uptake following exercise. After activation, this kinase phosphorylates many other proteins, thereby activating molecular pathways that are involved in ATP synthesis, GLUT4 translocation to the plasma membrane, and mitochondrial activity. As discussed previously, AMPK is a redox sensitive protein, in which cysteines 299 and 304 are potential targets of H_2_O_2_. The oxidative modification of AMPK cysteine residues can increase its kinase activity regardless of ATP depletion [60]. Antioxidant pre-treatment prevents both glucose uptake and AMPK activation during contractile activity [75], linking these three elements in a plausible mechanism that controls muscle glucose uptake. Furthermore, peroxynitrite can directly activate AMPK and increase glucose uptake via the insulin-independent translocation of GLUT4 [80]. Although some evidence suggest that AMPK coordinates the ROS-stimulates glucose uptake following muscle contraction and/or exercise, studies using a either a chronic [81] and an inducible [82] muscle-specific AMPK deletion did not confirm the involvement of AMPK activation in exercise-stimulated glucose uptake, showing that an uncharacterized redox sensitive signaling pathways may play a role in glucose uptake induced by exercise.

## 7. The Role of ROS in Physical Exercise Adaptations

Skeletal muscle is a specialized tissue with a remarkable plasticity. Successive muscle contractions at each exercise session led to a variety of physiological adaptations, which are closely related to the type of exercise (Figure 4). Physical exercise can be divided into two main types: endurance (aerobic resistance training) or muscular strength (anaerobic resistance training) [83]. The molecular machinery involved in physical exercise adaptations are closely related to the exercise type [1,84]. Moreover, an improvement in insulin sensitivity and muscle glucose uptake [9] is observed when both exercise programs are prescribed to individuals. These adaptations are reversible, and upon the absence of contracting stimulus, they return to their basal level. For example, 2 weeks of physical inactivity is enough to decrease learn body mass and impair insulin sensitivity [85]. Both responses to acute and long-term exercise are influenced by the volume, intensity, and frequency of the contractile stimuli along with the half-life of specific exercise-induced proteins [86].

The central player in endurance exercise adaptations is the PGC-1α [87,88]. Exercise is a powerful stimulus to induce PGC-1α mRNA and PGC-1α protein expression in rodents and humans [89,90,91,92]. PGC-1α has emerged as the mechanistic link to a wide range of beneficial effects of exercise in muscle, such as mitochondrial biogenesis, angiogenesis, fatty acid utilization, and fiber type switching [84,93], and antioxidant defense [94].

During exercise, there is an intense systemic adrenergic activation that is important for cardiovascular adjustments and substrate mobilization. Moreover, the increased sympathetic tone that is elicited by exercise upregulates muscle PGC-1α expression via β2-adrenergic receptor signaling [91]. In addition, muscle contraction also triggers other cellular signaling pathways that elevate PGC-1α mRNA levels, such as 5-adenosine monophosphate-activated protein kinase (AMPK) [95,96], p38 mitogen-activated protein kinases (p38 MAPK) [97], and Ca2+/calmodulin-dependent protein kinase (CaMK) [98]. Failure to stimulate PGC-1α expression in a chronically exercised mouse model leads to limited muscle physiological adaptations, highlighting the absolute requirement of PGC-1α for exercise training-induced changes in muscle mitochondrial biogenesis, angiogenesis, and fiber type changes [87,99,100,101]. Markedly, failure to stimulate many of these above-mentioned exercise effectors can be caused by a disrupted redox signaling that occurs during antioxidants supplementation (Figure 4).

In myotubes that are electrically stimulated, PGC-1α was shown to be induced by CREB signaling through O_2_^•−^ that are generated by NOX2 [38]. Moreover, treatment with allopurinol, an XO inhibitor, prevented the increase of PGC-1α and its target genes in the vastus lateralis muscle of rats after a single session of anaerobic exercise [102]. Lactate can stimulate mitochondrial biogenesis in cardiac and skeletal muscle [103], and recently, lactate-induced PGC-1α was shown to be blocked by antioxidant treatment, in C2C12 skeletal muscle cells [104], suggesting that a ROS can couple with lactate metabolism to stimulate PGC-1α production.

A truncated splice variant of PGC-1α called PGC-1α4, was shown to be induced by resistance but not by endurance training [105]. PGC-1α4 was able to increase IGF-1 but not the same set of oxidative genes such as TFAM, PGC-1α and NRF2, and its expression was associated with hypertrophic muscle response [105]. Therefore, current observations placed PGC-1α as a central player in orchestrating both endurance (oxidative) and resistance (myogenic) adaptations to exercise. However, it is not known whether redox-mediated signaling is involved in this alternative splice and PGC-1α4 expression.

### 7.1. Mitochondrial Biogenesis and Antioxidant Defense

The importance of NOX2 in many physiological adaptations induced by physical exercise responses has been reported [12,18]. Using a new NOX2-specific ROS production biosensor of human p47phox fused to the N terminus of redox-sensitive green fluorescent protein (p47roGFP), Henriquez-Olguin et al. showed that NOX2 levels were increased in skeletal muscle immediately after one physical exercise session, returning to basal levels after 1 h. The acute response to exercise related to antioxidant gene transcription, such as increased SOD2 and glutathione peroxidase, were blocked by the pharmacological inhibition of NOX2, as was the expression of mitochondrial biogenesis genes (e.g., TFAM and citrate synthases mRNA), suggesting that NOX2-derived O_2_^•−^ is involved in NF-κB and NRF-2 pathway activation that is elicited by physical exercise [106]. High-intensity interval training adaptations were examined, and a blunted response to physical exercise related to the maximal running capacity and some mitochondrial adaptations to exercise training (e.g., Complex I, III, IV and PDH levels) were shown in a mouse model with a non-functional NOX2 complex resulting from the absence of p47phox that was subjected to 6 weeks of training. Moreover, SOD2 and catalase induction were also shown to be dependent on NOX2 activation [30].

A switch of glycolytic into a more oxidative skeletal muscle fiber type was shown to be an important adaptation of endurance training that ameliorated exercise capacity [107]. Loureiro et al. showed that exercise training at 60% of the maximum run speed for 3 weeks increased NOX2 expression and activity in slow-twitch fibers, while NOX4 expression was increased only in the red portion of the gastrocnemius in rats. No changes were observed in the white portion of the gastrocnemius, probably due to the exercise protocol [32]. However, even under three different training protocols (voluntary running for 4 weeks or forced treadmill training for 10 days or 7 weeks) training-induced fiber switch was not affected by the absence of NOX4 in mice. Moreover, AMPK/PGC-1α activation also occurred in a NOX4-independent manner [108]. Interestingly, whole body and endothelial-specific NOX4 deletion disrupted some metabolic responses and adaptations to exercise in mice. Muscle glucose and fatty acid oxidation and UCP3 expression increase in response to acute exercise were blunted in those mice. When NOX4-deficient mice were subjected to 4 weeks of training, they showed an impaired time to exhaustion and a lack of induction of citrate synthase activity (a marker of mitochondrial adaptation to exercise). In addition, NOX4 seems to be crucial to promote upregulation of many genes involved in the early transcriptional responses after acute exercise (for instance, UCP3, PDK4, and HK2). However, exercise-dependent PGC-1α upregulation was not dependent on NOX4 expression [51], suggesting that a temporospatial regulation of NOX isoforms and/or even other sources of ROS control specific responses to exercise.

Furthermore, NOX4 seems to be crucial to the beneficial effect of physical exercise on cardiomyocyte antioxidant defense, which seems to be mediated by NOX4/NRF2 axis [109]. Acute exercise increases NOX4 but not NOX2 in the heart and cardiomyocytes, and NOX4-deficient mice presented a reduced exercise capacity that was associated with cardiac oxidative stress and mitochondrial dysfunction [109].

XO expression and activity is much more evident in endothelial cells than in skeletal muscle [110] (#1 in Figure 4), but it seems to play an important role in RONS-mediated signaling in the gastrocnemius muscle following an exhaustive exercise session in rats [52]. Allopurinol, an XO inhibitor, prevented the activation of P38 MAPK and ERK that was induced by exhaustive exercise, which was related to a lack of NF-κB activation, and consequently SOD expression [52]. Dual-specificity phosphatases (DUSPs) counteract MAPK activity and its Cys 258 might be involved in inactivating DUSP phosphatase activities and triggering their proteasomal degradation [111,112] (Figure 4). Wardley et al. analyzed the role of O_2_^•−^ that were derived from XO in acute and chronic exercise responses. They observed that XO inhibition attenuated some ROS-mediated signaling (e.g., P38 MAPK and ERK phosphorylation) in acute exercise, but it did not prevent the increase of PGC-1α, NRF2, GLUT4, and SOD mRNA levels and mitochondrial adaptation to exercise training [113].

### 7.2. Hypertrophy and Mass Regulation

IGF-1 that is produced locally by muscle cells, which act in an autocrine and paracrine manner, is believed to be the most important hypertrophic stimulus. IGF-1 post-transcriptional regulation creates distinct molecules, among which is mechano-growth factor (MGF) [114]. Both proteins are considered to be important mediators of exercise-induced skeletal muscle hypertrophy, which can occur through ROS signaling [115].

PI3K/AKT/mTOR pathway activation increases protein synthesis, but how this pathway is activated remains poorly understood [116]. Recently, a redox control of this pathway during exercise has been discussed [117]. mTOR phosphorylation is increased several hours after resistance training, preferentially in type 2 fibers [118]. Moreover, the hypertrophic response is attenuated by a mTOR inhibitor, rapamycin [119]. mTOR is also activated through the focal adhesion kinase (FAK). Mechanical strain on the sarcolemma activates FAK with consequent mTOR induction, which drives the mechanosensory pathway by activating P70S6K and consequently increasing protein synthesis [120].

During exercise-mediated hypertrophy signaling, phosphatase and tensin homolog (PTEN) and protein phosphatase 2A (PP2A) control two crucial steps in PI3K/Akt signaling and they can be targeted by H_2_O_2_-mediated signaling (Figure 4). Besides the transitory inhibition of PTEN [121], H_2_O_2_-mediated signaling causes phosphorylation of PTEN, triggering its degradation [122]. For PP2A, it has been demonstrated that peroxynitrite-mediated nitration resulted in the inhibition of PP2A, which promotes sustained activation of PI3/AKT signaling [123]. Additionally, AKT, alone, is also a sensitive redox protein that has a disulfide bond between Cys297 and Cys311 following treatment with H_2_O_2_, which is important for its association with PP2A, and consequently, Akt dephosphorylation and thereby inhibits AKT activation. However, the AKT redox state did not affect its kinase activity [124]. On the one hand, the redox status leads to an association between PP2A with AKT, while on the other hand, ROS-mediated signaling leads to an inhibition of PTEN and PP2A, which are two major regulators of PI3K/AKT signaling, therefore underlying the role of a pro-oxidant environment after exercise in sustaining the PI3K/AKT cascades to mediate hypertrophic stimuli.

It is noteworthy to point out that only few studies have aimed to investigate ROS-mediated cellular signaling following resistance exercise protocols (#2 in Figure 4). For example, the involvement of PI3/Akt as a redox sensitive cascade to promote hypertrophy is still unknown and furthers studies are needed (#4 in Figure 4).

## 8. Fine-Tuned Redox-Mediated Signaling Promotes the Beneficial Effects of Exercise in Muscle

It is currently thought that fine-tuned redox regulation is necessary to reach exercise-induced physiological adaptations, and external factors, such as antioxidant ingestion, can act to hamper the ROS-dependent effects of exercise in muscle. Several studies demonstrated that ROS scavengers impair training-induced improvements in the maximal exercise capacity in rodents and humans [125,126] and prevents the increment of lean body mass after resistance training [127]. In addition to the maximal exercise capacity, another desirable response is the increase in muscular glucose uptake [128]. A diet supplemented with vitamins C and E prevented the improvement in insulin sensitivity in both untrained and pre-trained healthy young men followed by reduced PPARα, PGC-1α and PGC-1b expression [10].

Recently, new insights have been identified about an endogenous factor in obesity, which disrupts ROS-mediated signaling following exercise. About 20% of obese people are unresponsive to physical exercise programs and do not benefit like other people [129]. SEPP1 is a hepatokine that is involved in selenium (Se) transport to many tissues such as brain, kidney, and muscle, which is crucial for the biosynthesis of selenoproteins such as thioredoxin and glutathione peroxidase (GPX) [130] and can act as a potent antioxidant because of its cysteine residues. Glucocorticoids, inflammation, and obesity directly increase SEPP1 expression in hepatic cells, which is closely related to its increase in blood levels [131]. In an elegant study, Misu et al. demonstrated that SEPP1 could communicate the liver to muscle through the upregulation of GPX1, which maintained a reduced intracellular environment that was related to a lack of ROS-induced AMPK activation and PGC-1α following physical exercise. Finally, the authors suggest that SEPP1 inhibitors may function as exercise-enhancing drugs to treat diseases that are associated with a sedentary lifestyle [131]. Takamura recently pointed to SEPP1 as the first endogenous factor capable of promoting “reductive stress” during exercise by hampering the physiological ROS bursts that are required for the beneficial effects of exercise [132].

The notion that antioxidants disrupt the AMPK cascade after exercise was also demonstrated in a study where antioxidant ingestion prevented Thr286 CaMK phosphorylation and activation, contributing to the subsequent decrease in Thr172 AMPK phosphorylation and its activation [71]. Moreover, antioxidant ingestion was also shown to increase the phosphorylation of Ser485 from AMPKa1 and Ser491 from AMPKa2, which were responsible for abrogating AMPK activation by preventing the stimulatory phosphorylation at Thr172 [133]. AKT-mediated Ser phosphorylation was shown to prevent AMPK activation even with increased AMP levels [133,134,135]. Antioxidant ingestion prevented exercise induced Thr172 phosphorylation of AMPK, even when the AMP/ATP ratio is changed as a result of exercise. AMPK signaling is involved in exercise-induced upregulation of GLUT4 and its translocation to the membrane. Thus, this may be the link between a reduced exercise training improvement in insulin sensitivity when antioxidants are administered [10] (Figure 4 box inside).

This effect suggests that decreasing ROS-mediated signaling via either exogenous antioxidants (e.g., intake of vitamin C/E) or endogenous SEPP1 upregulation is able to negatively affect exercise-induced AMPK activation in the muscle. Beyond the positive effect of exercise on skeletal muscle cells, other tissues are also responsive to exercise through a fascinating and orchestrated network between contracting muscle and remote tissues. Interestingly, increased ROS generation and biomolecules oxidation after exercise are found in remote tissues such as heart [136], liver [137], brain [138] and white adipose tissue [139]. One hypothetical model that fits well with the widespread health benefits of exercise is the generation of regular and synchronized ROS waves in contracting muscle and then in remote tissues, which lead to a transient prooxidative state followed by redox mediated signaling and cytoprotection [3].

Antioxidant protein levels and activities seem to be fiber-specific, and generally an increase in their activities occurs in response to one section and training protocols [140,141]. One session of physical exercise increases the SOD content and activity in both slow- and fast-twitch fibers [142]. However, GPX activity increased only in slow-twitch fibers [143]. The important role played by endogenous antioxidant defense to ensure the fine-tuning of redox signaling following exercise adaptations was highlighted using Mn-SOD-deficient animals, in which the absence of exercise adaptations was observed in these Mn-SOD-deficient animals only when high intensities were used [144]. Although mitochondrial antioxidant defense was absent in this previous study, deleterious signaling was not observed during excessive ROS generated by high-intensity exercise, suggesting that non-interconnected mechanisms are involved in generating large amounts of ROS during exercise and in response to deleterious stimuli, such as what is observed in immobilization [145,146] and cancer induced muscular atrophy [147] and muscular disorders following aging [148]. The intricate complexity of how ROS-mediated signaling either leads to healthy adaptations or to maladaptive and muscle dysfunction remains controversial. For example, muscle denervation causes atrophy because of higher mitochondrial ROS generation [149]. This suggests that even when ROS generation is higher, as observed following strenuous exercise, it does not mean that an atrophy mechanism is likely to be observed in skeletal muscle [150,151], suggesting that a detailed understanding of these different roles played by the “ROS that are able to promote a beneficial effect” compared to “ROS that are able to promote a harmful effect” is an important and emerging area in redox biology and muscle function [3,145,146,147,148,152].

This concept can be easily observed during muscle glucose uptake where muscle NOX2 is placed paradoxically in a delicate position regarding glucose homeostasis. In vitro experiments show that insulin-stimulated O_2_^•−^ generation through NADPH mediates the activation of the intracellular Ca2+ increase, exemplifying the dependency between insulin action and NOX-dependent ROS-mediated signaling [153]. Paradoxically, diabetic challenges (e.g., high glucose and palmitate exposure) impair insulin sensitivity, and this also occurs via up-regulation of O_2_^•−^ production from NADPH oxidases [154] and from mitochondrial sources [155]. The double-edged sword effect of muscle NOX2 can be also observed in vivo, where NOX2-generated ROS can be either implicated in the beneficial effects of exercise in glucose homeostasis [24,30] or it can negatively affect muscle insulin sensitivity in a high fat diet mouse model [154].

## 9. Conclusions

Some of the physical exercise adaptations seem to occur after sequential exercise-induced ROS and are explained by the concept of hormesis, in which a potentially harmful agent provides an adaptation to a damaging agent (from a RONS disrupted signaling) and increasing, for example, antioxidant capacity. Current data are continuously demonstrating a permissive effect of ROS-mediated signaling in driving many of the most important signaling pathways that occur without an elevation in oxidative stress markers. Fine-tuned redox mediated signaling is required to correctly activate the best-known molecular effectors following exercise (e.g., antioxidant capacity, mitochondrial biogenesis, hypertrophy, and improvement of insulin sensitivity). However, it is too early to accurately provide the main source of ROS behind the ROS-mediated signaling, but recent studies have shown a crucial role of NOX2-derived O_2_^•-^ activating several intracellular cascades that are involved in physical exercise responses.

Unless there is a disruption in redox mediated signaling that is derived from a pathological condition, healthy exercise ROS production is undeniably required to promote the beneficial effects of exercise. Understanding the reasons and limits between “beneficial effects” and “harmful effects” of ROS is one of the most challenging issues raised in physical exercise biology. Finally, the identification of new mediators that disrupt exercise-stimulated ROS signaling and hamper the beneficial effects of exercise may allow the identification of specific targets for therapeutic intervention.
